# The Regulatory Network of MicroRNA in the Metabolism of Colorectal Cancer

**DOI:** 10.7150/jca.61618

**Published:** 2021-11-04

**Authors:** Wangji Li, Yan Lu, Changda Ye, Manzhao Ouyang

**Affiliations:** 1 Department of Gastrointestinal Surgery, Shunde Hospital, Southern Medical University (The First People's Hospital of Shunde Foshan), Shunde, Foshan, Guangdong Province, 528300, China.; 2 The Second School of Clinical Medicine, Southern Medical University, Guangzhou, Guangdong Province, 510080, China.

**Keywords:** colorectal cancer, intestinal flora, metabolism, microRNA, molecular biomarkers, non-coding RNA, regulatory network, therapeutic targets

## Abstract

Colorectal cancer (CRC) is the third most common malignant tumor in the world. During the progression of CRC, the entire metabolic network undergoes reprogramming, including marked changes in the regulation of glucose, lipid and amino acid metabolism. Although microRNAs (miRNAs) account for only 1% of the entire human genome, they play an important role in almost all physiological and pathological processes in the body. MiRNAs can react directly with key enzymes in the metabolic processes. MiRNAs also interact with other ncRNAs, as a member of non-coding RNA (ncRNA), to form their own regulatory network in various oncogenic pathways of CRC metabolism. The progression of colorectal cancer is closely related to the intestinal flora, where miRNAs act as important mediators. Understanding how miRNAs act in the regulatory network of CRC metabolism is helpful to elucidate the characteristics of tumor occurrence, proliferation, metastasis and drug resistance. This review summarizes the role of miRNAs in the metabolism of CRC and how miRNAs interact with key enzymes, ncRNA and intestinal flora to further discuss how miRNAs affect CRC and realize some new strategies for the early diagnosis and treatment of CRC.

## Introduction

Colorectal cancer is the second leading cause of cancer-related death worldwide and its incidence is increasing yearly 1,2, which is a multi-step, multi-stage and multi-gene involved cytogenetic disease 3. In the progression of colorectal cancer, many factors contribute to dysregulation of the CRC signal pathway that promote tumor spread, metastasis and cause drug resistance 4. The onset of CRC is insidious, and definitive diagnosis usually relies on colonoscopic biopsy and related tumor markers. Tumor markers play a role in heralding tumor recurrence or metastasis but lack specificity. Currently, the principal treatment options for CRC include surgical resection, radiotherapy, chemotherapy and immunotherapy. Improvements in these treatment methods have significantly impacted and enhanced the prognosis of CRC, especially in early-stage disease. Even with the notable advances in the management of CRC, further progress is both achievable and necessary. Therefore, it is important to search for molecular markers that can more effectively detect tumor progression, but perhaps even more importantly, understand their role in CRC and their specific actions in their regulatory networks. The metabolism of CRC mainly includes glycolysis, lipid metabolism and amino acid metabolism. The metabolic reprogramming of CRC represents changes made by tumor cells to adapt to the environment. And the key enzymes and signaling pathways involved in the metabolic pathways have become a hot topic in CRC study.

**Figure 1:**
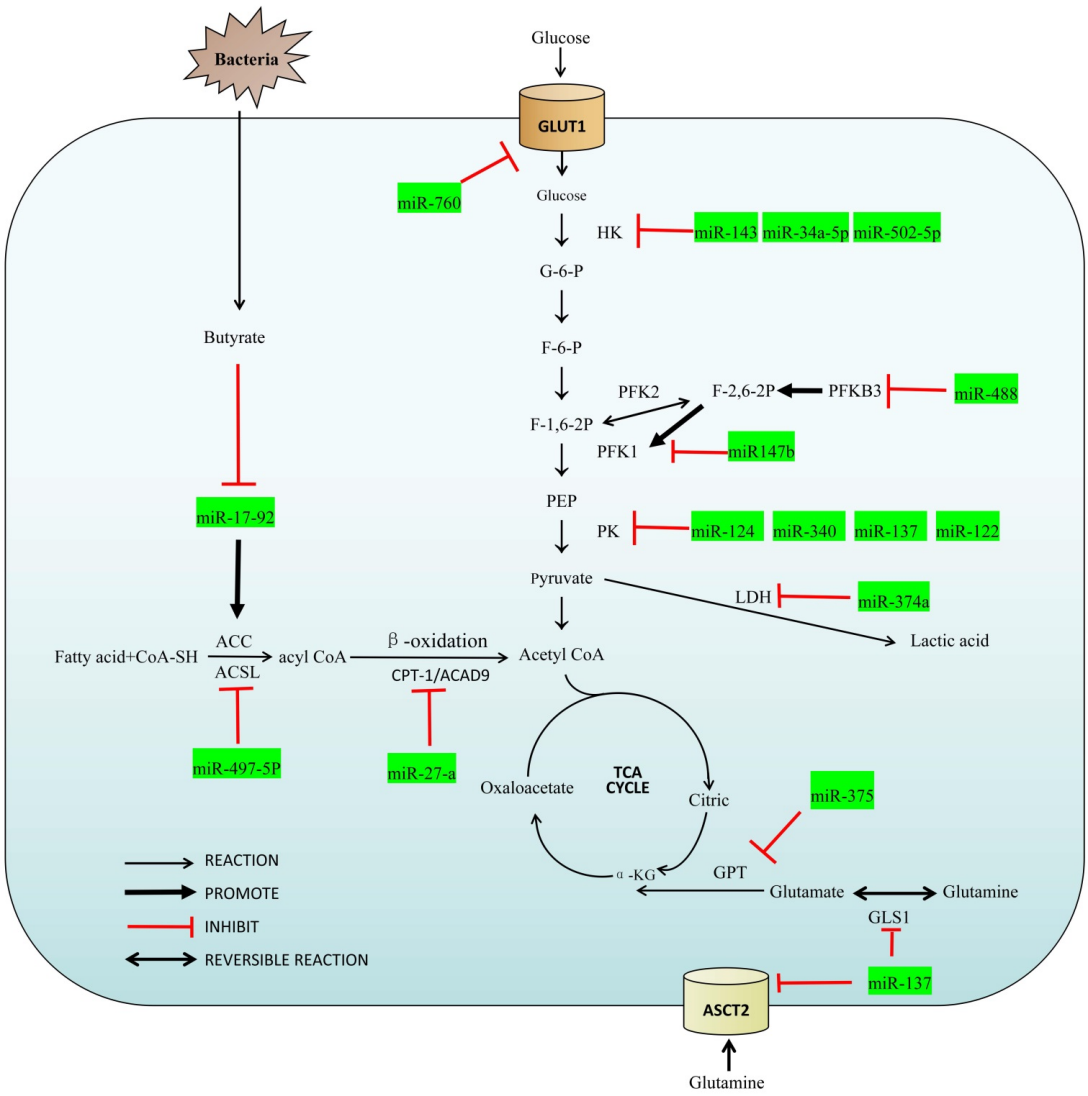
Overview of the miRNAs in the regulatory network of colorectal cancer.

MicroRNAs, members of the non-coding RNA family, are single-stranded RNA molecules of about 22 nucleotides in length. Although miRNAs account for only 1% of the entire human genome, they play an important role in the regulatory network for colorectal cancer 5,6. They can be coupled completely or incompletely by miRNA response elements (MRE) to the 3' untranslated region (3' UTR) of the target mRNA that drives the RNA induced silencing complex (RISC) to degrade the target mRNA, and inhibit expression of the target mRNA at the post-transcriptional level 5,7. MiRNAs can also interact with other ncRNAs to form their own regulatory effects in various oncogenic CRC pathways 8. In addition, we also summarized the relationship between miRNA and intestinal flora metabolism, which is a special part of colorectal tumors. With this overview of their influence on metabolic pathways, a more in-depth description of these and how miRNAs can be utilized in the molecular diagnosis, treatment and postoperative monitoring. A summary of the miRNAs and their target enzymes as well as other molecular interactions are shown in Figure 1 and Table 1 respectively.

## MicroRNA and glucose metabolism

Metabolic reprogramming is a hallmark of malignancy, and treatment resistance and tumor metastasis are facilitated as a consequence of these altered pathways 7. In the metabolic reprogramming of colorectal cancer, the abnormal performance of glucose metabolism is particularly prominent. Cancers still conduct glycolysis, but at a rate 200 times that of normal cells. This is necessary because cancers rely principally on glycolysis for energy production, yielding only 2 ATP, a rather inefficient method of energy production 9,10. In contrast, normal cells finish the process (Krebs cycle and the electron transport chain) that generates about 36 ATP. This dramatic reliance on aerobic glycolysis is named the Warburg effect 11,12, and is seen in almost all solid tumors cells. Current research regarding miRNA on the basis of this abnormal tumor-related aerobic glycolysis may provide new ideas for the early diagnosis and therapies of colorectal cancer 13. CRC regulates the uptake and utilization of glucose by adjusting the activity of glucose metabolism-related receptors or enzymes 14. Following, we summarize enzymes and transporter involved in the regulation of miRNA during glucose metabolism.

### Glucose transporter 1

Glucose transporter 1 (GLUT1) is a transmembrane protein on the cell membrane and a key transporter for the metabolism of glucose in mammalian cells, which is usually up-regulated in tumor cells 15. Extracellular glucose cannot pass freely through the cell membrane, it must be entered into the cell with the help of GLUT1 to participate in glucose metabolism. Santasusagna et al. 16 found that miR-328 can bind to the SLC2A1-3'UTR and reduce the level of GLUT1 through SLC2A1/ GLUT1 pathway in CRC. Zhang et al. 17 found that GLUT1 was the target of miR-760, and the level of GLUT1 increased when miR-760 was decreased. Both miR-328 and miR-760 are negative correlation with the level of GLUT1 so that the up-regulate of these miRNAs can consequently down-regulate GLUT1-mediated glycolytic activity in CRC.

### Hexokinase

The function of Hexokinase (HK) is to convert glucose into G-6-P, which is a rate-limiting enzyme in glycolysis. There are four important subtypes of mammalian hexokinase, named HK1 to HK4. In particular, HK2 is dysregulated in a variety of cancers including CRC and is often found upregulated in CRC as to adapt to the high rate of glucose metabolism necessary 18-20. Gregersen et al. 21 found miRNA-143 can target the HK2-3'UTR to reduce its expression in colorectal cancer. Liu et al. 22 found miR-502-5p inhibited the expression of HK2 and Li et al. 23 found miR-34a-5 inhibited the expression of HK1. These miRNAs are all impeded aerobic glycolysis in CRC by inhibit the expression of HK and act as a tumor suppression factor. Chen et al. 24 found low expression of miR-513a-3p in CRC cells increased HK2 expression and promoted proliferation and metabolism in colorectal cancer cells.

### Phosphofructokinase-1

The function of Phosphofructokinase-1 (PFK-1) is to convert G-6-P into fructose-1,6-bisphosphate (F-1,6-2P) and it is also a rate-limiting enzyme in glycolysis. The function of Phosphofructokinase-2 (PFK-2) is to convert G-6-P into fructose-2,6-bisphosphate (F-2,6-2P) which is a strong activator of PFK-1. PFKFB3, a protein expressed by pfkfb3 gene and often highly expressed in CRC, can promote the synthesis of F-2,6-2P. Deng et al. 25 found PFKFB3 may be downstream of miR-488 and is negatively regulated by miR-488. The increasing expression of PFKFB3 can reverse the inhibition of miR-488 on glycolysis suggesting that the miR-488/ PFKFB3 axis may stimulate FPK1 by influencing F-2,6-2P, and ultimately affect the glycolysis process.

### Pyruvate kinase

Pyruvate kinase (PK) is to covert phosphoenolpyruvate (PEP) to pyruvate and it is the third rate-limiting enzyme in glycolysis including PKM1 and PKM2. PKM1 is expressed in most well-differentiated tissues and mainly promotes oxidative phosphorylation. PKM2, however, is only expressed in rapidly proliferating cells such as embryonic cells and cancer cells, and can promote glycolysis even under aerobic conditions 26-28. Sun et al. 29 and Clower et al. 30 found that miR-124, miR-137 and miR-340 could down-regulate the selective splice protein acting on the PKM gene (PTB1/hnRNAPA1/ hnRNAPA2), thereby promoting the conversion of PKM2 to PKM1. High ratios of PKM1/PKM2 can inhibit aerobic glycolysis so that miR-124, miR-137 and miR-340 can act as regulator of glucose metabolism in CRC. Huang et al. 31 found that CuET, as a metabolite of an alcoholism treatment drug DSF, is capable of regulating the miR-16-5p, miR-15b-5p/ALDH1A3/PKM2 axis. CuET inhibits ALDH1A3, an important isoform of the aldehyde metabolic enzyme system ALDH family, by selectively enhancing expressions of miR-16-5p and miR-15b-5p and then inhibits the ubiquitination of PKM2, so as to alleviate the glycolysis of CRC cells.

### Lactate dehydrogenase

Lactate dehydrogenase (LDH) is to convert pyruvate to lactic acid, lactate levels in cells are also an important factor regulating glycolysis. Janney et al. 32 found that LDHA expression in CRC, one isoform of LDH, might have a negative correlation with miR-34a, miR-34c, miR-369-3p, miR-374-a and miR-4524-a/b expression. The 3'UTR region of LDHA relative gene can match with miR-374a. When miR-374a is overexpression in CRC, the decrease of LDHA cells will lead to the accumulation of pyruvic acid. Thus, miRNA regulate glycolysis by affecting the conversion of pyruvate to lactic acid.

## MicroRNA and lipid metabolism

Lipid metabolism, an important and complex biochemical reaction in the body, is the process of digestion, absorption, synthesis and decomposition of lipid in cells. Lipid metabolism not only provides energy, but lipids are also an integrate component of the cell membrane. MiRNAs frequently act on enzymes in lipid metabolism, are related to the proliferation and progression of tumor cells, so the regulatory network for lipid metabolism is summarized below.

### Acyl-CoA synthetase

Fatty acids must be activated before being oxidized to generate acyl-coenzyme A (acyl CoA) under the catalysis of Acyl-CoA synthetase (ACS). ACLS5, one of the subtypes of ACS, is highly expressed in colorectal cancer and has been proved to be regulated by miRNA^33^. Gharib et al. 34 found that miR-497-5p can target the ACSL5-3'UTR and reduce its expression in colorectal cancer so that fatty acids cannot synthesize acyl-CoA normally, resulting in abnormal β-oxidation. MiR-497-5p reduces the energy produced by colorectal cancer from lipid metabolism by inhibiting ACSL, suppressing the development of CRC. This has also stimulated a new treatment strategy: the starvation therapy.

### Carnitine palmitoy transterase-1 and Acyl-CoA Dehydrogenase 9

Carnitine palmitoy transterase-1 (CPT-1) and Acyl-CoA Dehydrogenase 9 (ACAD9) are the two important enzymes in β-oxidation. Barisciano et al. 35 found that CPT-1 and ACAD9 may be potential targets for miR-27a. When colorectal cancer cells have miR-27a knockout, the levels of both proteins increased; in contrast, both proteins decreased with overexpression of miR-27a. Of note, the effect of miR-27a on glucose metabolism was reviewed previously, and it can also regulate lipid metabolism in CRC 36. Thus, the regulation of miR-27a on metabolic reprogramming can affect tumor invasiveness and drug resistance in CRC.

## MicroRNA and amino acid metabolism

Amino acid metabolism has been a hot spot in cancer research recently. When tumor cells proliferate and synthesize proteins rapidly, a large number of essential and non-essential amino acids are required 37. In the metabolic regulation network of CRC, glutamine is an important molecule. It can be converted into α-ketoglutaric acid (α-KG) to enter the tricarboxylic acid cycle (TCA cycle, Krebs cycle) as a key replenishment component of the TCA. Similarly, α-KG is also a raw material for the synthesis of nucleotides, fatty acids and other non-essential amino acids 38. Several enzymes and the glutamine transporter participate in this process are regulated by miRNA.

### ASC family transporter 2

ASC family transporter (ASCT) is a glutamine transporter, which mainly transports extracellular glutamine into cells and participates in amino acid metabolism. Among them ASC family transporter 2 (ASCT2, also known as SLC1A5) is generally up-regulated in human cancers and involved in metabolic reprogramming 39. Dong et al. 40 found that the level of ASCT2 in most tumor cells, including CRC cells, is regulated by miR-137. MiR-137 can directly target the ASCT2-3'UTR, resulting in down-regulation of ASCT2 expression, thereby reducing the uptake of glutamine in CRC.

### Glutaminase 1

Glutaminase 1 (GLS1) is an enzyme that catalyzes the formation of glutamate from glutamine. It has been previously noted that miR-137 can inhibit ASCT and, in addition, it can also reduce the GLS1 levels. Li et al. 41 found that miR-137 may be a GLS1-targeting miRNA, regulated by HSF1. The miR-137 can be inhibited by HSF1 because of promoter methylation of the miR-137 upstream gene mir137hg that then reduces expression of miR-137. Inhibition of GLS1 by miR-137 therefore inhibits the decomposition and transformation of glutamine. Because glutamine is very important to energy supply and molecular synthesis, miR-137 reduced the growth of CRC cells through this regulatory network.

### Glutamic pyruvic transaminase

Glutamic pyruvic transaminase (GPT) converts glutamate to α-ketoglutaric acid, which is a critical component of the TCA. CRC cells with mutations in pik3ca have a higher requirement for glutamine due to the catalytic subunit P110α. This change results in an increase in the expression of glutamic pyruvic transaminase 2 (GPT2) as an adaptation to the energy requirement for the rapid growth and proliferation of CRC 42. The miR-375 can bind to the PIK3CA-3'UTR and inhibit the expression of PIK3CA in CRC, thereby inhibiting its growth through the PI3K/Akt pathway 43. The lower expression of PI3K leads to a consequent decrease in the expression of GPT2, thus limiting the utilization of glutamine in CRC.

## MicroRNA and other non-coding RNA

Non-coding RNAs can be linked to the regulation network of tumor cell metabolism, proliferation, metastasis and apoptosis through miRNA 8. These regulatory non-coding RNAs include small non-coding RNA (sncRNA), long non-coding RNA (lncRNA), and circle RNA (circRNA). Some lncRNAs and circ RNAs act as competitive endogenous RNAs (ceRNA) to affect the post-transcriptional regulation of genes by binding to miRNA directly. They usually have miRNA binding sites and play a role of miRNA sponges to produce the corresponding biological effects 44-46. A deep understanding of the regulatory network for non-coding RNA interactions may help to elucidate the role of miRNA in the regulatory network and provide a new direction for the prevention, early diagnosis and therapy of CRC. The following section focuses on the role of these ncRNAs and their interaction in the lncRNA-miRNA-mRNA regulatory network.

### MicroRNA and lncRNA

**Figure 2:**
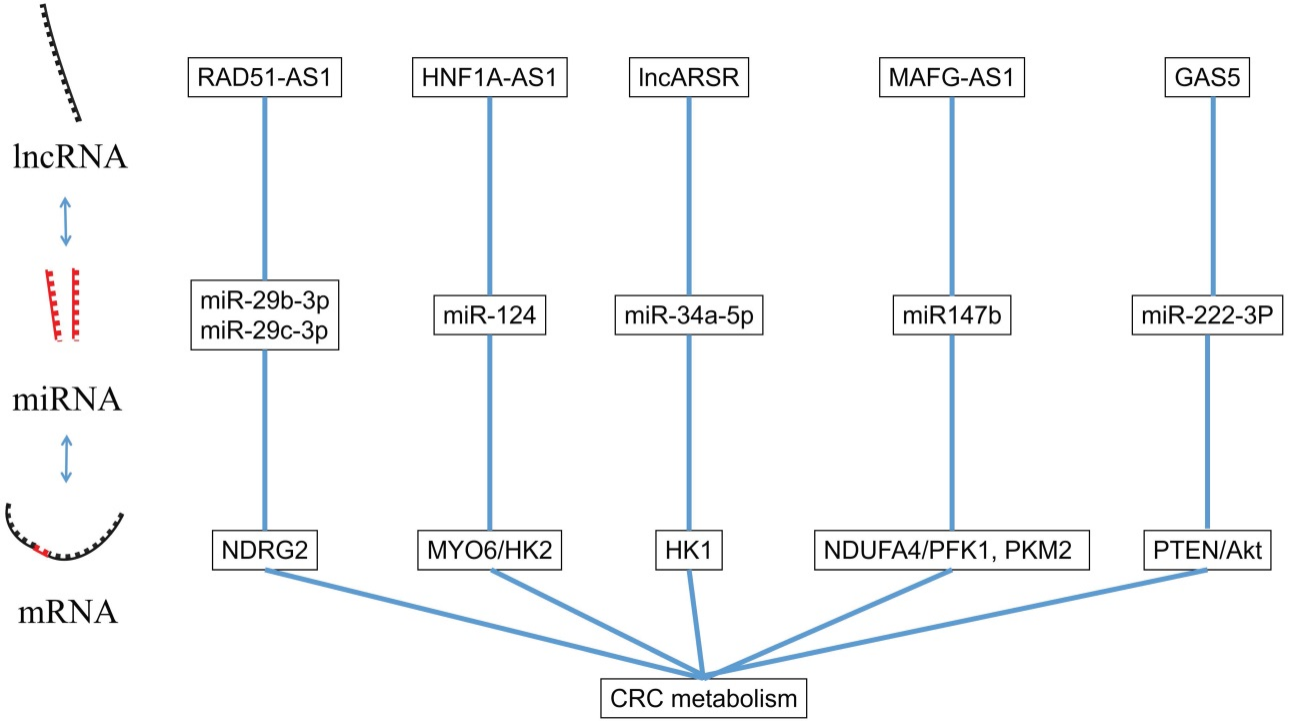
The interaction between miRNA and lncRNA.

LncRNA is a kind of RNA molecule with a length of 200~100000nt and its regulation in colorectal cancer is varied 47. LncRNA regulates the level of chromatin in the nucleus 48, and also regulates the transcription of its adjacent mRNA through the cis-acting element 49. In the following sections, we will summarize several lncRNAs involved in the proliferation, apoptosis, invasion and migration of CRC (Figure 2).

#### LncRNA RAD51-AS1

The dysregulation of LncRNA RAD51-AS1 has been reported as closely related to a variety of cancers 50. Li et al. 51 found that LncRNA RAD51-AS1 acts as a tumor suppressor in CRC, yet LncRNA RAD51-AS1 can also affect the glycolysis of CRC by affecting HK2 and GLUT. Furthermore, LncRNA RAD51-AS1 can bind to miR-29b-3p and miR-29c-3p through sponge effect and up-regulates NDRG2, which has been reportedly involved in the regulation of glycolytic metabolism 52. LncRNA RAD51-AS1 exerts its tumor suppressor effect through miR-29b/c-3p/NDRG2, and inhibits the proliferation, invasion and aerobic glycolysis of CRC.

#### Hepatocyte nuclear clear factor receptor 1 homeobox A-antisense RNA 1

In colorectal cancer tissues and cell lines, the expression of lncRNA hepatocyte nuclear clear factor receptor 1 homeobox A-antisense RNA 1 (HNF1A-AS1) was significantly upregulated 53. Guo et al. 54 found that miR-124 was a target of HNF1A-AS1, and MYO6 was a target mRNA of miR-124 in CRC cells. The reduction of HNF1A-AS1 or up-regulation of miR-124 resulted in a similar biological effect: inhibition of migration and invasion of CRC cells and glycolysis. Further experiments demonstrated that down-regulation of miR-124 promoted expression of MYO6, which was positively associated with HK2 in CRC cells. Therefore, HNF1A-AS1 can influence cell migration, invasion, and glycolysis by regulating the miR-124/ MYO6 axis of colorectal cancer cells.

#### LncARSR and MAFG-AS1

Li et al. 23 found that lncARSR could promote the expression of HK1 in colorectal cancer through target inhibition of miR-34a-5p, and Cui et al. 55 found that lncRNA MAFG-AS1 could promote the expression of PFK1 and PKM2 in colorectal cancer through the miR-147b/NDUFA4 axis. These lncRNAs can regulate the key enzymes of glycolysis in CRC through miRNA.

#### Growth arrest-specific 5

Liu et al. 56 found that lncRNA growth arrest-specific 5 (GAS5) can competitively bind to miR-222-3P, which can target the PTEN-3'UTR gene and inhibit its expression. PTEN is an anti-oncogene gene which was reported to be related to cancer cell proliferation and migration. The tumor suppressor protein expressed by PTEN can dephosphorylate Akt and reduce its activation. It is a negative regulator of the PI3K/Akt pathway which can prevent all downstream signaling events regulated by Akt 57. Therefore, lncRNA GAS5 led to the inactivation of Akt through miR-222-3P/ PTEN axis and affected the proliferation and migration of CRC.

### MicroRNA and circRNA

**Figure 3:**
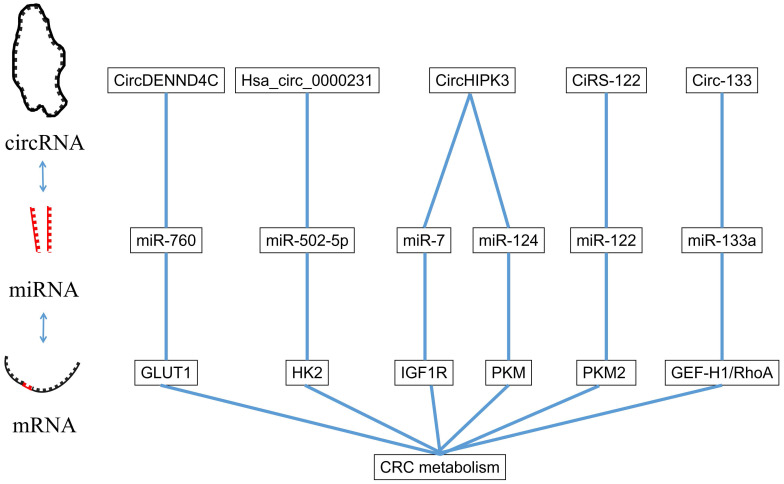
The interaction between miRNA and circRNA.

CircRNA is a kind of special RNA molecule with a closed ring structure, without either a 5'-end or 3'-end. It is not affected by RNA exonuclease and its expression is more stable and it is not easily degraded 58. The biological function of circRNA in the human body was gradually revealed with the development of high-throughput sequencing technology. The circRNA acts as a ceRNA to target the downstream gene of miRNA. In the following sections, we will summarize several circRNAs involved in the proliferation, apoptosis, invasion and migration of CRC and the pathway they involve (Figure 3).

#### CircDENND4C

We have demonstrated that miR-760 can act as a regulator in the glycolysis by targeting the GLUT1, and circDENND4C can also take part in the miR-760/GLUT1 axis. Zhang et al. 17 proved that circDENND4C could target miRNA-760, reduce its inhibitory effect on GLUT1 by the action of a miRNA sponge, and finally promote glycolysis in CRC cells.

#### Hsa_circ_0000231

MYO6 is a key substance that links circRNA, miRNA and glucose metabolism in CRC. Huang et al. 59 found that Hsa_circ_0000231 could compete with miR-502-5p as a ceRNA. The miR-502-5p can promote the expression of MYO6 which is positively correlated with HK2 in CRC cells. So Hsa_Circ_0000231 acts on MYO6 through miR-502-5p to regulate glycolysis in colorectal cancer.

#### CircHIPK3

Transcription factor c-Myb regulates metabolism, proliferation, and metastasis in CRC through a variety of signaling pathways 60. It has been shown that c-Myb can improve the transcription of circHIPK3 in colorectal cancer 61-63. Zeng et al. 61 found that miR-7 could inhibit the expression of insulin-like growth factor receptor 1 (IGF1R) in CRC cells, whereas overexpression of circHIPK3 could effectively reverse the protective effect of miR-7 in CRC. IGF1R mediates metabolic changes, cell proliferation, and invasion and metastasis of colorectal cancer cells by activating PI3K/Akt pathway, which has been reported to be related to cancer cell metabolism, proliferation and migration. Zheng et al. 62 found that circHIPK3 could also competitively inhibited miR-124, thereby inhibiting the PKM2 to PKM1 transition, ultimately promoting glycolysis in CRC. Zhang et al. 63 found that circHIPK3 inhibits autophagy in CRC cells by competitively binding to miR-637, activating the STAT3 signaling pathway, increasing Bcl-2 expression, and blocking Beclin-1 expression, resulting in increased drug resistance to oxaliplatin based chemotherapy.

#### CiRS-122

CircRNA is widely present in exosomes due to its structural stability and achieves connection to the regulatory network between CRC cells through its miRNA sponge effect. Wang et al. 64 found that exosomes from colorectal cancer cells that have developed oxaliplatin resistance can promote glycolysis in chemotherapy-sensitive cells and reduce their drug sensitivity. These exosomes contain ciRS-122, which can be transmitted to sensitive cells to inhibit miR-122 through the miRNA sponge effect. The miR-122 can bind to the PKM2-3'UTR and reduce PKM2 expression, resulting in upregulation of glycolysis, thus producing resistance to oxaliplatin.

#### Circ-133

Oxygen supply in CRC is unbalanced, resulting in altered metabolic balance within tumor cells, and the development of hypoxic and normoxic cancers 65. This difference results in an environment that is not conducive for hypoxic cancer cells to proliferate and grow. However, a strong regulatory network for colorectal cancer appears to alter this condition. Yang et al. 66 found that tumor cells derived from an environment of low partial pressure of oxygen, were able to secrete exosomes containing circ-133. These exosomes promoted the invasion and metastasis of tumor cells by acting on the miR-133a/GEF-H1/RhoA signaling pathway in tumor cells derived from normal partial pressure of oxygen. This intercellular regulation redistributes oxygen resources in the TME and promotes the invasion and metastasis of CRC.

## MicroRNA and intestinal flora metabolism

The development of colorectal cancer is closely related to the intestinal flora 67. High levels of dietary fibre have been reported to reduce the risk of colon cancer in humans and animal models 68, attributed to short-chain fatty acids (SCFA) 69,70. Butyrate is one of the major components of SCFAs, involved in the metabolism of normal colorectal epithelial cells, and it is an important component of energy metabolism 71,72. In CRC cells, most of the butyrate is not metabolized due to the Warburg effect. The accumulated butyrate can act as a histone deacetylase inhibitor (HDAC), which is involved in regulating the metabolism, proliferation and apoptosis of tumor cells 73.

### miR-17-92

Hu et al. 74 found that butyrate can decrease the expression of c-Myc and hinder the combination of c-Myc and E-box elements (E3) in the core promoter region, resulting in a decreased expression of miR-17-92. Izreig et al. 75 found that miR-17-92 binds to the 3'-UTR region of tumor suppressor gene liver kinase B1 (LKB1). LKB1 can rapidly inactivate acetyl-CoA carboxylase (ACC) and HMG-coenzyme A reductase (HMGCR) by activating AMP-dependent protein kinase (AMPK), thus inhibiting the synthesis of fatty acids and cholesterol in tumor cells, but also slightly upregulating the activity of PFK-1 and promoting glycolysis 76. Thus, butyrate from the intestinal flora can remodel the metabolic regulatory network of CRC through negative regulation of the c-Myc/miR-17-92 axis, reduce its energy source overall, resulting in inhibition of tumorigenesis.

### miR-106b

In addition, butyrate can also play an anti-tumor role by regulating the proliferation, metastasis and apoptosis of colorectal cancer through miRNA. p21 is a cyclin dependent kinase (CDK) inhibitor located downstream of p53. It can form a G1 checkpoint in the cell cycle with p53, thus reducing the replication and accumulation of damaged DNA, thereby exerting an anti-cancer effect 77. Hu et al. 78 found that butyrate increased p21 protein expression by inhibiting miR-106b.

### miR-203

HEF1 is a focal adhesion protein that proved to be important in the regulation of the CRC cell. Han et al. 79 found that miR-203 inhibits butyrate by promoting miR-203 expression, thereby prolonging the colorectal cancer cell cycle by inhibiting HEF1 and tumor cell colony formation. Although butyrate has been shown to have excellent antitumor effects, its short half-life and low bioavailability still limit its clinical application.

## Conclusions and perspectives

Colorectal cancer is the third most common tumor worldwide, the second leading cause of cancer-related death, and its incidence is increasing yearly. It is valuable and becoming increasingly necessary to have a comprehensive understanding of miRNAs in the CRC regulatory network. In the review, we summarize the role and regulation of CRC-related miRNAs in the metabolism and the molecular interaction between miRNAs and other ncRNAs. Additionally, the regulatory network of miRNAs in intestinal flora metabolism is also included.

The research of miRNAs has been carried out for many years and has made excellent progression in drug application and biomarkers for rapid diagnosis. However, the research on its molecular mechanism is still relatively insufficient. Regarding the research on the mechanism of the regulatory network in miRNA and CRC metabolism is mostly based on the complete or incomplete complementary pairing with the mRNA of related metabolic genes after the miRNA binds to the AGO protein to form a RISC complex, thereby mediating its degradation or inhibiting translation. However, this mechanism is applicable mainly to miRNAs distributed in the cytoplasm. Other mechanisms in CRC metabolisms, such as interactions with other RNA binding proteins, negative regulation of RNA precursor transcripts, combine with the promoter in regulation transcription levels and mediate the distribution of RNA inside and outside the nucleus. However, the current research on this area is still not deep enough. More molecular mechanisms of miRNA in CRC metabolism need to be further elucidated.

In clinical treatment, the diagnosis of CRC mainly relies on biopsy under colonoscopy and the treatment is mainly surgery, supplemented by radiotherapy, chemotherapy, and targeted therapy. Although the accuracy of pathological diagnosis is very high at present, the surgical treatment of colorectal cancer is becoming more and more mature in technology, and there are still some patients who cannot benefit from it. Lack of specific diagnostic methods due to its insidious symptoms, many CRC patients are already in the advanced stage at the time of diagnosis. Drug therapy is the primary treatment for unresectable stage and extensively metastasized patients, but tumor drug resistance makes the treatment effect in some patients poor. Most studies have mentioned that non-coding RNA has great potential in tumor diagnosis and drug treatment. However, according to the clinical trial database of NIH (ClinicalTrials.gov), there are only a few items about CRC-related ncRNA trials, indicating the inadequacy in clinical translational. Non-coding RNA is an ideal choice as a marker for early diagnosis of CRC due to its stability, specificity and non-invasiveness of detection methods. It is essential to promote non-coding RNA research transform into clinical trials or clinical applications.

At the same time, research on new uses for existing drugs is also eye-catching. It seems to be a short-term solution to the current clinical dilemma due to the long development cycle of new anti-tumor drugs. Metabolism reprogramming is common in various tumors. Since metformin and some traditional nonsteroidal anti-inflammatory drugs (NSAIDs) have also been found anti-tumor effects by affecting tumor metabolism. Recently, the anti-alcohol metabolism drug DSF has also been found can affects the glycolysis of tumors by targeting miRNAs, which provides new ideas for non-coding RNA research.

Therefore, from the perspective of the miRNA regulatory network, exploring the metabolic reprogramming mechanism of CRC may be able to solve the problem of poor specificity and resistance of antimetabolites in the treatment of CRC, but there is still a long way to go.

## Figures and Tables

**Table 1:** List of miRNAs involved in metabolism and its molecular interaction of CRC

MicroRNA	Expression in CRC	Target/Pathway	Description	Effect on CRC	Sample	Refs
miR-143	↓	HK2	Inhibit aerobic glycolysis	Proliferation	Cell line	21
miR-513a-3p	↓	HK2	Inhibit aerobic glycolysis	Proliferation	Cell line	24
miR-488	↓	PFKFB3/PFK-1	Inhibit aerobic glycolysis	Proliferation, migration and invasion	CRC tissues and cell line	25
miR-124	↓	PKM2, PKM1	Inhibit aerobic glycolysis, inhibited by lncRNA HNF1A-AS1 and circHIPK3	Cancer growth	Cell line	29,62
	↓	MYO6/HK2		Migration and invasion	CRC tissues, paracancerous tissues and cell line	54
miR-137	↓	PKM2, PKM1	Inhibit aerobic glycolysis Suppress glutamine uptake and utilization	Cancer growth	Cell line	29,40
	↓	ASCT2, GLS1		Tumorigenesis	Cell line	
miR-340	↓	PKM2, PKM1	Inhibit aerobic glycolysis	Cancer growth	Cell line	29
miR-16-5p/miR-15b-5p	↑	ALDH1A3/PKM2	Inhibit aerobic glycolysis	Cancer growth and apoptosis	Cell line	31
miR-374a	↓	LDHA	Suppress lactate production	Cell growth and tumorigenesis	Cell line	32
miR-497-5p	↓	ACSL5	Suppress fatty acyl-CoA production	Proliferation, metastasis and invasion	CRC tissues and cell line	34
miR-27a	↑	PGC-1α, PPARγ, CPT1A and ACAD9	Inhibit fatty acids oxidation	Proliferation, migration, invasion and drug resistance	Cell line	35
miR-375	↓	GPT2 via PI3K/Akt pathway	Suppress glutamine utilization	Cancer growth	Cell line	42,43
miR-34a-5p	↓	HK1	Inhibit aerobic glycolysis, can be inhibited by lncARSR	Invasion and metastasis	Cell line	23
miR-147b	↓	NDUFA4, PFK-1, PKM2	Inhibit aerobic glycolysis, can be inhibited by lncRNA MAFG-AS1	Proliferation	Cell line	55
miR-222-3P	↑	PTEN/PI3K/Akt	Promote metabolic reprogramming, can be inhibited by IncRNA GAS5	Migration and invasion	Cell line	56
miR-760	↓	GLUT1	Suppress glucose transport, can be inhibited by circDENND4C	Proliferation and migration	Cell line	17
miR-502-5p	↓	MYO6/HK2	Inhibit aerobic glycolysis, can be inhibited by Hsa_circ_0000231	Proliferation, migration and invasion	CRC tissues, paracancerous tissues and cell line	22
miR-455-3p	↓	MYO6	Inhibit aerobic glycolysis, can be inhibited by circCSNK1G1	Cell growth and metastasis	CRC tissues, paracancerous tissues and cell line	59
miR-7	↓	IGF1R via PI3K/Akt pathway	Inhibit metabolic reprogramming, can be inhibited by circHIPK3	Proliferation, migration and invasion	Cell line	62
miR-637	↓	Beclin-1 via STAT3 pathway	Inhibit the autophagy of tumor cell, can be inhibited by circHIPK3	Cell growth and apoptosis	CRC tissues, paracancerous tissues and cell line	63
miR-122	↓	PKM2	Inhibit aerobic glycolysis, can be inhibited by ciRS-122	Apoptosis	Cell line	64
miR-133a	↓	GEF-H1/RhoA	Promote tumor invasion and metastasis, can be inhibited by circ-133	Metastasis	Cell line	66
miR-17-92	↑	ACC, HMGCR via LKB1/AMPK pathway	Promote the synthesis of fatty acids and cholesterol, can be inhibited by butyrate	Proliferation	Cell line	74,75
miR-106b	↑	p21	Promote the proliferation of tumor cell, can be inhibited by butyrate	Proliferation	Cell line	78
miR-203	↓	HEF1	Inhibit tumor invasion and metastasis, can be promoted by butyrate	Proliferation, invasion, apoptosis	Cell line	79

## Abbreviations

CRC: Colorectal cancer
miRNA: microRNA
GLUT1: Glucose Transporter 1
CuET: Diethyldithiocarbamate-copper complex
DSF: Disulfiram
HK: Hexokinase
HSF1: heat shock factor 1
PFK-1: Phosphofructokinase-1
F-1,6-2P: fructose-1,6-bisphosphate
PFK-2: Phosphofructokinase-2
F-2,6-2P: fructose-2,6-bisphosphate
PK: Pyruvate kinase
PEP: phosphoenolpyruvate
LDH: Lactate dehydrogenase
ACS: Acyl-CoA synthetase
acyl CoA: acyl-coenzyme A
CPT-1: Carnitine palmitoy transterase-1
ACAD9: Acyl-CoA Dehydrogenase 9
α-KG: α-ketoglutaric acid
ASCT: ASC family transporter
GLS1: Glutaminase 1
GPT: Glutamic pyruvic transaminase
sncRNA: small non-coding RNA
lncRNA: long non-coding RNA
circRNA: circle RNA
ceRNA: competitive endogenous RNAs
HNF1A-AS1: hepatocyte nuclear clear factor receptor 1 homeobox A-antisense RNA 1
GAS5: growth arrest-specific 5
ZEB: zinc finger protein
IGF1R: insulin-like growth factor receptor 1
SCFA: short-chain fatty acids
LKB1: liver kinase B1
ACC: acetyl-CoA carboxylase
HMGCR: HMG-coenzyme A reductase
AMPK: AMP-dependent protein kinase
CDK: cyclin dependent kinase
